# New insights into laticifers in *Mimosa caesalpiniifolia* Benth. (Fabaceae): anatomy, ultrastructure, and development, emphasizing the presence of callose

**DOI:** 10.1007/s00709-026-02179-w

**Published:** 2026-03-03

**Authors:** Tatiane Maria Rodrigues, Stefany Cristina de Melo Silva, Yasmin Massimino Sampaio de Souza, Lúcio Queiroz de Carvalho, Yve Canaveze, Silvia Rodrigues Machado

**Affiliations:** 1 Department of Biodiversity and Biostatistics, Institute of Biosciences of Botucatu, São Paulo State University (UNESP), Professor Antônio Celso Wagner Zagnin street, 250, District of Rubião Júnior, Botucatu City, 18618-970 São Paulo State Brazil; 2 Interunit Postgraduate Program in Plant Biology, Institute of Biosciences of Botucatu and Rio Claro, São Paulo State University (UNESP), Botucatu, Brazil; 3 Department of Botany, Federal University of São Carlos (UFSCar), São Carlos City, São Paulo State Brazil

**Keywords:** Articulated nonanastomosing laticifers, Callose, Leguminosae, Phloem, Wall thickenings

## Abstract

Laticifers are specialized structures found in diverse plant families and are regarded as important components of plant defense systems. In Fabaceae, however, laticifers are relatively uncommon and have been reported in only a few genera, including *Mimosa*. Despite this, little is known about the ultrastructural features of laticifer protoplasts and cell walls in this group, as well as their distribution throughout the plant body. This study investigated the distribution, anatomy, and ultrastructure of laticifers in *Mimosa caesalpiniifolia* Benth. (Fabaceae: Caesalpinioideae) from a developmental perspective, encompassing the embryo, seedling, and adult stages. Articulated nonanastomosing laticifers were found associated with both primary and secondary phloem. Total lipids, acidic polysaccharides, and terpenes were detected within the laticifer protoplasts. Primordial laticifer cells displayed thick pectocellulosic walls containing plasmodesmata, dense cytoplasm and abundant organelles. Young laticifers exhibited digitiform extensions toward neighboring cells, while in maturing laticifers, the terminal walls of aligned cells underwent dissolution. Mature laticifers showed an additional discontinuous internal parietal layer of uniform appearance, composed of callose. To the best of our knowledge, this study provides the first report of callose in laticifers of a legume species. The potential protective role of callose as a self-cytotoxic barrier and as a defense mechanism against natural enemies in the laticifer system is hypothesized. This study fills an important gap in the knowledge of laticifer origin and typology in Fabaceae and provides valuable insights to support taxonomic, ecological, and sustainable-use studies of this multipurpose forest species native to the Brazilian semi-arid region.

Main Conclusion. Articulated, nonanastomosing laticifers are present throughout the development of *Mimosa caesalpiniifolia*, occurring in mature embryos,seedlings, and adult plants. A discontinuous parietal layer composed of callose internally lines the laticifer cells.

## Introduction

Laticifers are isolated or interconnected cells that form a system permeating the plant body and secrete a fluid known as latex, which is composed of various classes of chemical substances (Evert 2006). Latex produced by several plant species has traditional and contemporary economic and medicinal significance (Hagel et al. 2008). It is crucial in the production of rubber and drugs for medicinal and recreational use (Pickard 2008). Latex corresponds to the cytoplasm produced by laticifer cells (Freitas et al. 2024) constituted by a colloidal suspension of small particles in an aqueous sap (Abarca et al. 2019). The isolation of bioactive compounds within laticifers may protect the plant against the self-cytotoxic effects of these substances (Hagel et al. 2008). Latex stored in laticifers is released in response to injury (Ramos et al. 2019), and plays an important role in defense against herbivores and pathogens and in wound healing (Fahn 1979; Farrell et al. 1991; Freitas et al. 2024).

The cytological features of laticifers vary. In some species, the protoplasts of laticifers remain intact after maturation, whereas in others, the nuclei and organelles are degraded. In laticifers, the vacuome is composed of either multiple small vacuoles or vesicles, or a large central vacuole. In some species, the tonoplast disappears as a result from autophagy process and the lumen of the laticifer cells become filled with latex particles (see Evert 2006). The thickness, structure, and composition of laticifer walls also vary (Fahn 1979). In addition to pectocellulosic compounds, suberin and callose have been detected in laticifer walls (Fineran et al. 1988; Medina et al. 2021), in addition to lignin (Carlquist 1996). However, the presence of primary pit fields with plasmodesmata in laticifer walls remains controversial.

Laticiferous species have been recorded in more than 40 phylogenetically distant families, predominantly in tropical regions (Prado and Demarco 2018). Most species with laticifers belong to the eudicotyledons, particularly Apocynaceae, Euphorbiaceae, Cannabaceae, Moraceae and Sapindaceae (Demarco and Castro 2008; Lopes et al. 2009; Canaveze et al. 2019; Leme et al. 2020; Medina et al. 2021; Marques et al. 2022; Naidoo et al. 2023; Figueiredo et al. 2025; Mouzella et al. 2025), and more rarely to the legume species (Lewinsohn 1991; Fernadez-Dattoli et al. 2011; de Oliveira et al. 2021). The presence of laticifers and their morphological and functional characteristics are of great taxonomic importance, with the occurrence of these structures being diagnostic for some families (Simpson 2010).

Fabaceae represents one of the largest families of angiosperms, with approximately 745 genera and 19,500 species (LPWG 2017), many of which have great economic and ecological significance. The presence of laticifers among legumes appears to be an uncommon feature that has been reported in only a few species. Unbranched articulated laticifers have been observed in the phloem of *Lonchocarpus lilloi* leaves (Fernadez-Dattoli et al. 2011). In *Swartzia* species, articulated laticifers responsible for the production of reddish latex have been observed in the phloem of the stem bark (de Oliveira et al. 2021). Within the genus *Mimosa*, the occurrence of latex has been reported for *M. laticifera* (Lewinsohn 1991). However, the ultrastructural aspects of laticifer protoplasts and cell walls, as well as their distribution throughout the plant body, remain poorly understood.

*Mimosa caesalpiniifolia* Benth. (Fabaceae and Caesalpinioideae) is a native woody species that occurs mainly in northern and northeastern Brazil, although it is also widely distributed across the country. It is a heliophytic, pioneer, and selective xerophyte species that is widely used for the recovery of degraded areas and as a forage species. Preparations using its bark are used to treat bronchitis, stop bleeding, and wash wounds. Stem wood is rich in tannins, which can be used as adhesives for gluing wood (Carvalho 2007). When injured, its leaves and young stems release milky fluid, which was not observed in the stems of the secondary structure (personal observation). The abundance of latex released by the young organs, together with the scarcity of records of laticifers in species of the genus, motivated us to investigate in detail the sites responsible for the production of this milky secretion. Despite the ecological and economic importance of this species, no studies have been conducted on the distribution and structural aspects of laticifers in *M. caesalpiniifolia*.

This study aimed to characterize the distribution of laticifers in the embryos, seedlings, and adult plants of *M. caesalpiniifolia*, in addition to characterizing their structure, histochemistry, and developmental cytology.

## Materials and methods

### Plant material

*M. caesalpiniifolia* plants possess alternate, bipinnate compound leaves with approximately four to six pairs of elliptical to oval leaflets and stipules. White, milky secretion (latex) was observed in the young stems and leaves after injury.

Adult plants (n = 5) growing in a rural area in Botucatu, São Paulo, Brazil (22°53’ S 48°26’ W) were sampled. Vouchers (registration number 30405) were deposited in the BOTU Herbarium “Irina Delanova Gemtchújnicov” of the Institute of Biosciences, UNESP, Botucatu.

Samples of the shoot apex, stem in primary structure, stem in secondary structure, young leaves, and expanded leaves were collected using a heated razor (Canaveze and Machado 2016) to reduce latex leakage at the time of injury.

To obtain mature embryos, seeds were obtained from mature fruits after their dehiscence. The seeds were soaked in water for 3 h and their coats were then removed to collect mature embryos (*n* = 10). Seeds were also placed in acrylic boxes lined with filter paper and kept moist with distilled water in germination chambers at 25 °C until the eophylls expandede. Samples were collected from the median regions of the cotyledon, eophyll, hypocotyl, and primary root of the seedlings (*n* = 10). The seedling phase was defined as the phase between the protrusion of the primary root and the expansion of the first eophyll.

### Light microscopy

Samples were fixed in Karnovsky’s solution (Karnovsky 1965) for 24 h. Some samples were manually sectioned using a razor blade. Sections were stained with astra blue and safranin (Bukatsch 1972) and mounted between slides and coverslips using glycerin. Another portion of the material was dehydrated in a graded ethanol series, embedded in historesin (Leica Historesin^®^) and sectioned on a semi-automatic rotary microtome (Leica, RM2145). The 5-µm-thick transverse and longitudinal sections were stained with 0.05% Toluidine Blue, pH 4.2 (O’Brien et al. 1964) and the slides were mounted with synthetic resin.

For the histochemical detection of major classes of chemical compounds in laticifer cell walls and protoplasts, fresh samples were manually sectioned using razor blades and treated with different reagents: Sudan IV for total lipids (Johansen 1940); 0.02% Ruthenium Red solution for polysaccharides and pectin (Jensen 1962); Lugol’s reagent for starch grains and alkaloids (Johansen 1940); 10% aqueous ferric chloride solution for phenolic substances (Johansen 1940); acidified phloroglucinol for lignin (Sass 1951); Bromophenol Mercury Blue solution for protein (Mazia et al. 1953); Nadi reagent for terpenes/oil-resin (David and Carde 1964); Periodic Acid/Schiff Reagent (PAS) for neutral polysaccharides (Taboga and Vilamaior 2001); Wagner’s reagent for alkaloids (Furr and Mahlberg 1981); Tannic acid/Iron III chloride for mucilage (Pizzolato and Lillie 1973); Oil Red O for rubber particles (Jayabalan and Shah 1986). Control tests were performed according to the indications of the descriptors of each technique.

The material was analyzed under a photomicroscope (Olympus, BX 41), and the relevant results were documented with an attached digital camera (Olympus, C7070).

Alternatively, for detection of callose, hand-cut sections of fresh material were treated with 0.05% aniline blue solution in 0.067 M potassium phosphate buffer pH 8,5 for 5 min (Smith and McCully 1978). Autofluorescence was examined in sections not treated with aniline blue and mounted in 0.067 M potassium phosphate buffer pH 8,5. Samples were then analyzed using a confocal laser scanning microscope (Leica SP5), with emission range of 460–470 nm.

### Transmission electron microscopy (TEM)

Samples from the shoot apex (*n* = 3) and median region of the young stem (*n* = 3) obtained from adult plants were fixed in 2.5% glutaraldehyde and 0.1 M phosphate buffer (pH 7.3), post-fixed in 1% osmium tetroxide in the same buffer, then dehydrated in an acetone series, and embedded in Araldite resin (Machado and Rodrigues 2004). Ultrathin sections (90 nm) were treated with lead citrate and uranyl acetate (Reynolds 1963) and analyzed using a transmission electron microscope (Tecnai Spirit, FEI Company, USA) at 60 kV.

## Results

### Laticifer distribution

**Fig. 1:**
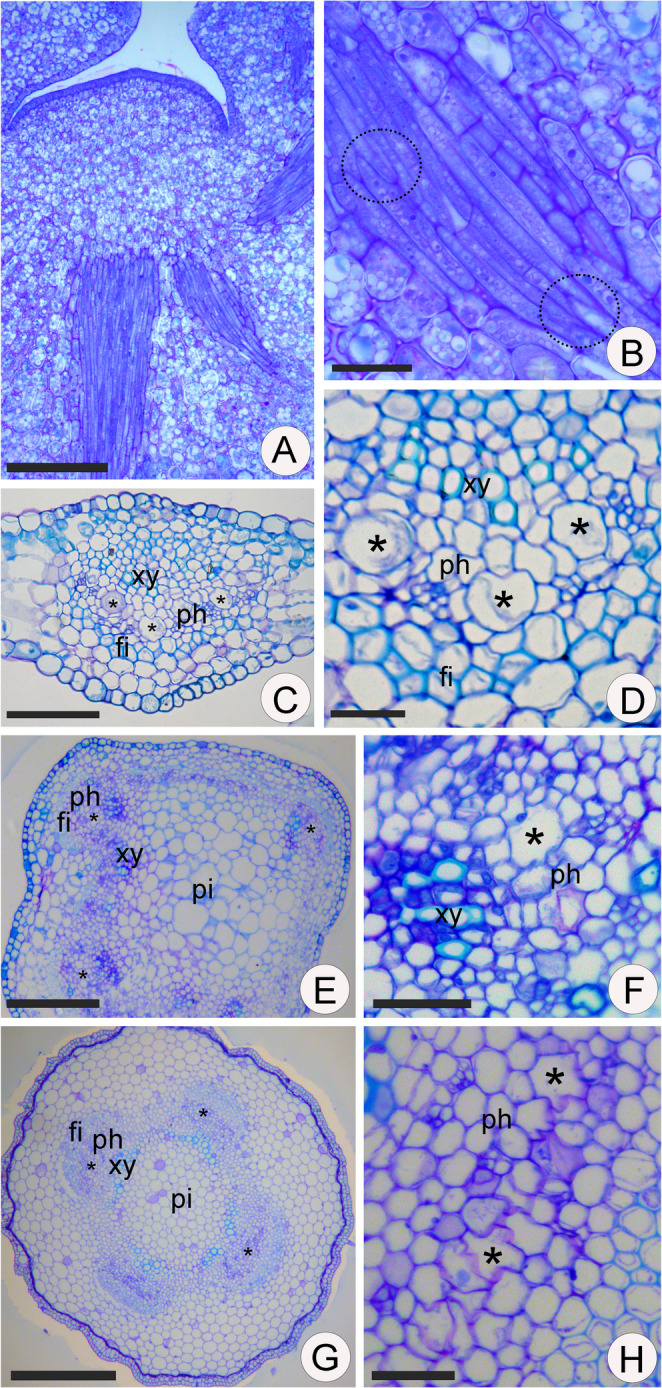
Light micrographs of mature embryo and seedling organs of *Mimosa caealpiniifolia* stained with toluidine blue. **A** Longitudinal sections of the upper portion of the embryonic axis. **B** Detail of the previous figure showing differentiating laticifers with acuminated tips (encircled areas) immersed in procambial strands. **C**,** D** Cross sections of eophyll. **E**,** F** Cross sections of epicotyl. **G**,** H.** Cross sections of hypocotyl. The * indicate laticifers. fi: pericyclic fibers, ph: phloem, pi: pith, xy: xylem. Scale bars: A, E, G = 200 μm; B, F, H = 50 μm; C = 100 μm

Laticifers were observed from the embryo to adult stage and were always associated with the phloem. In the mature embryo, differentiating laticifers were associated with procambial strands in the hypocotyl-radicle axis and were recognized by their thicker walls and serial arrangement (Fig. 1A, B). The protoplasts were dense and multinucleated (Fig. 1B). Some laticifers exhibited irregular outlines and acuminated and thick tips indicative of intrusive growth (Fig. 1B).

**Fig. 2:**
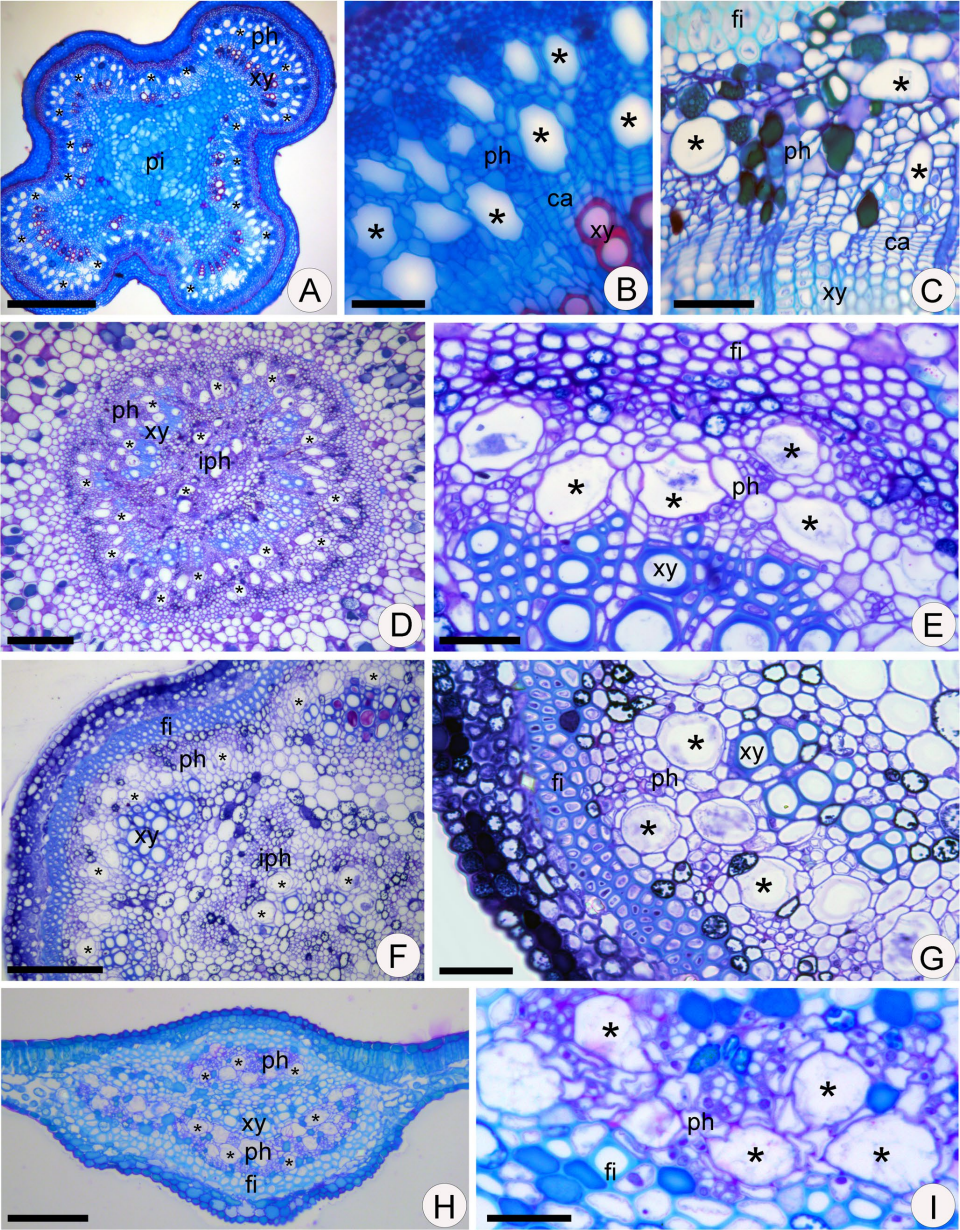
Light micrographs of stem and leaves of adult plants of *Mimosa caesalpiniifolia* in cross sections stained with astra blue and safranin (**A**,** B**) and toludine blue (**C-I**). **A**,** B** Stem in primary growth stage. **C** Stem in secondary growth stage. **D**,** E** Primary pulvinus. **F**,** G** Petiole. **H**,** I**. leaf midrib. The * indicate laticifers. ca.: vascular cambium, fi: pericyclic fibers, iph: inner phloem, ph: phloem, pi: pith, xy: xylem. Scale bars: A = 500 μm; B, C, H = 100 μm; D, F = 200 μm; E, G = 50 μm; I = 20 μm

In seedlings, laticifers were present in the eophyll (Fig. 1C, D), epicotyl (Fig. 1E, F) and hypocotyl (Fig. 1G, H). In the seedling organs, laticifers were apparently less voluminous and scarcer than in adult plants (Fig. 2).

In adult plants, laticifers were found in the stems in primary (Fig. 2A, B) and secondary (Fig. 2C) growth stages. In the secondary phloem of the stem (Fig. 2C), the laticifers were much less abundant than in the primary phloem (Fig. 2B) and were intermixed with phenolic idioblasts (Fig. 2C). In the leaves, laticifers were found in the pulvini (Fig. 2D, E), petioles (Fig. 2F, G), rachis, midrib (Fig. 2H, I), and veins immersed in the mesophyll. In pulvini (Fig. 2D), petioles (Fig. 2F) and rachis, laticifers occurred in both the outer and inner phloem portions of the bicollateral vascular bundles.

### Structural features of laticifers and developmental cytology

Laticifers at various developmental stages were located in procambial strands just below the promeristematic region of the stem. Four developmental stages of laticifers were identified based on the ultrastructural and light microscopic features of the secretory cells.

**Fig. 3:**
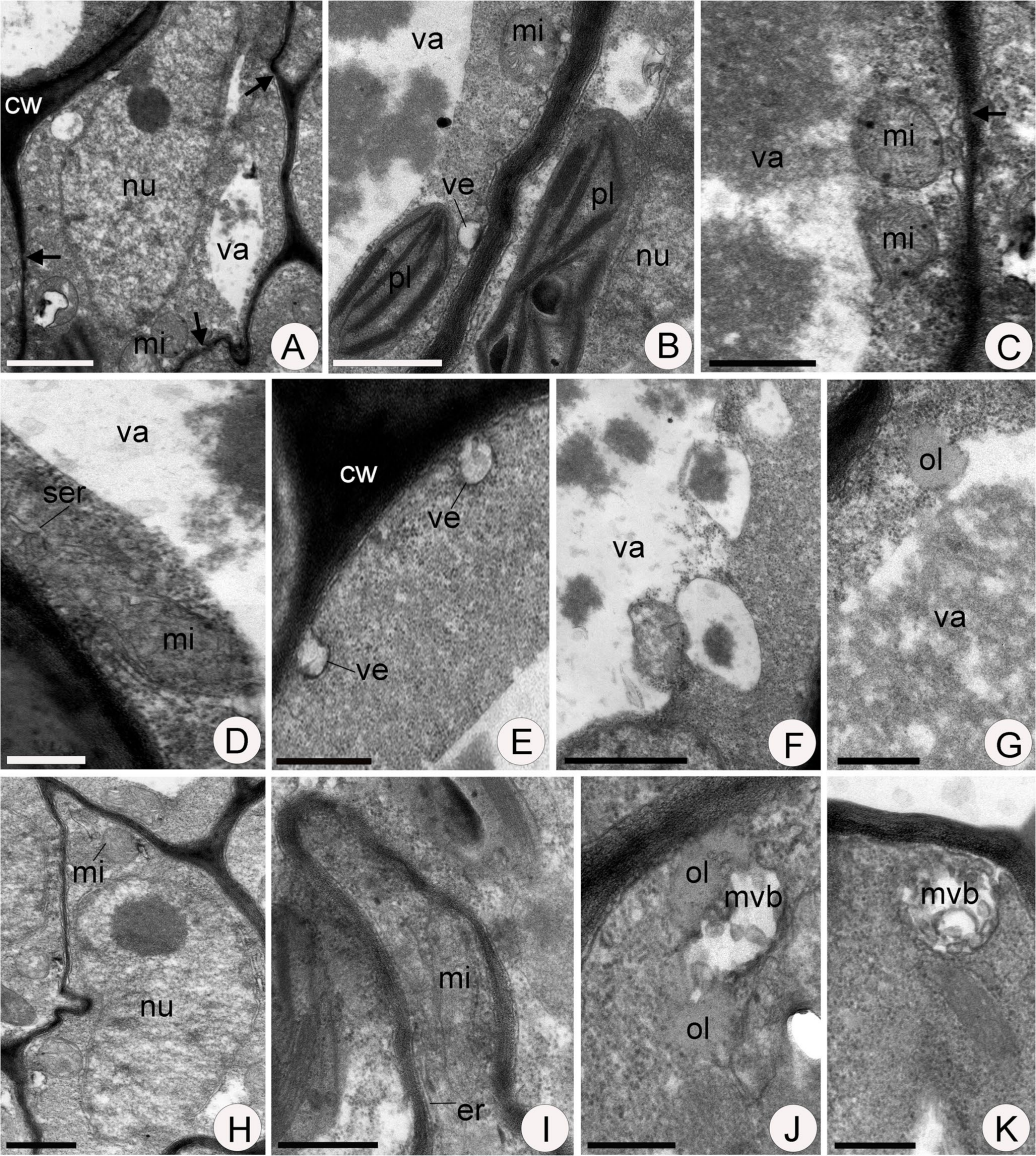
Transmission electron micrographs of *Mimosa cesalpiniifolia* laticifer cells in developmental stage 1. **A** Primordial cells with digitiform projections. Observe the dense cytoplasm and the large nucleus. **B** Chloroplasts with poorly developed thylakoids. **C** Detail showing sinuous plasmalemma and vacuole with flocculent content. **D** Smooth endoplasmic reticulum and mitochondria in the cytoplasm. **E** Vesicles with attached to the plasmalemma. **F** Provacuoles with flocculent content attached to the tonoplast. **G** Oil drop free in the cytoplasm. **H** Cell with apical growth exhibiting digitiform projection. **I** Detail of digitiform projection with dense cytoplasm. **J** Multivesicular body and oil inclusions in the cytoplasm. **K** Multivesicular body attached to the plasmalemma. The arrows indicate plasmodesmata. cw: cell wall, er: endoplasmic reticulum, mi: mitochondria, mvb: multivesicular body, nu: nucleus, ol: oil, pl: plastid, ser: smooth endoplasmic reticulum va: vacuole, ve: vesicle. Scale bars: A, C, E, G = 1 μm; B, F, H = 2 μm; D, J = 0.5 μm; I = 0.7 μm; K = 0.6 μm

Stage 1 laticifers were represented by primordial cells. These cells were linearly arranged identified by their relatively thick and dense pectocellulosic walls and their commonly pointed ends (Fig. 1B). These cells exhibited irregular contour (Fig. 3A), sinuous plasmalemma (Fig. 3B, C) and were connected to each other by plasmodesmata (Fig. 3A, C). In the stem portions closest to the apex, the laticifer cells had a dense cytoplasm and conspicuous, elongated nuclei with well-defined nucleoli (Fig. 3A). Chloroplasts with poorly developed thylakoids and small starch grains (Fig. 3B), mitochondria with dense globular inclusions (Fig. 3C), smooth endoplasmic reticulum (Fig. 3D), and abundant ribosomes (Fig. 3C-G) were observed in the cytoplasm. Vesicles and vacuoles contained flocculent material (Fig. 3A-F). Vesicles occurred attached to both the plasmalemma (Fig. 3E) and tonoplast (Fig. 3F). At this developmental stage, laticifers were characterized by apical growth (Fig. 3H), with digitiform projections extended toward neighboring cells (Fig. 3I). The digitiform projections had irregularly thickened cell walls with a loose appearance and regions of electron-dense material (Fig. 3I). Inside the digitiform projections, the cytoplasm was dense with abundant organelles (Fig. 3I). Lipid bodies were commonly observed in the cytoplasm (Fig. 3G, J). Multivesicular bodies (MVBs) were commonly seen in this developmental stage and occurred close to the plasmalemma when in contact with the transverse walls of developing cells (Fig. 3J, K).

**Fig. 4:**
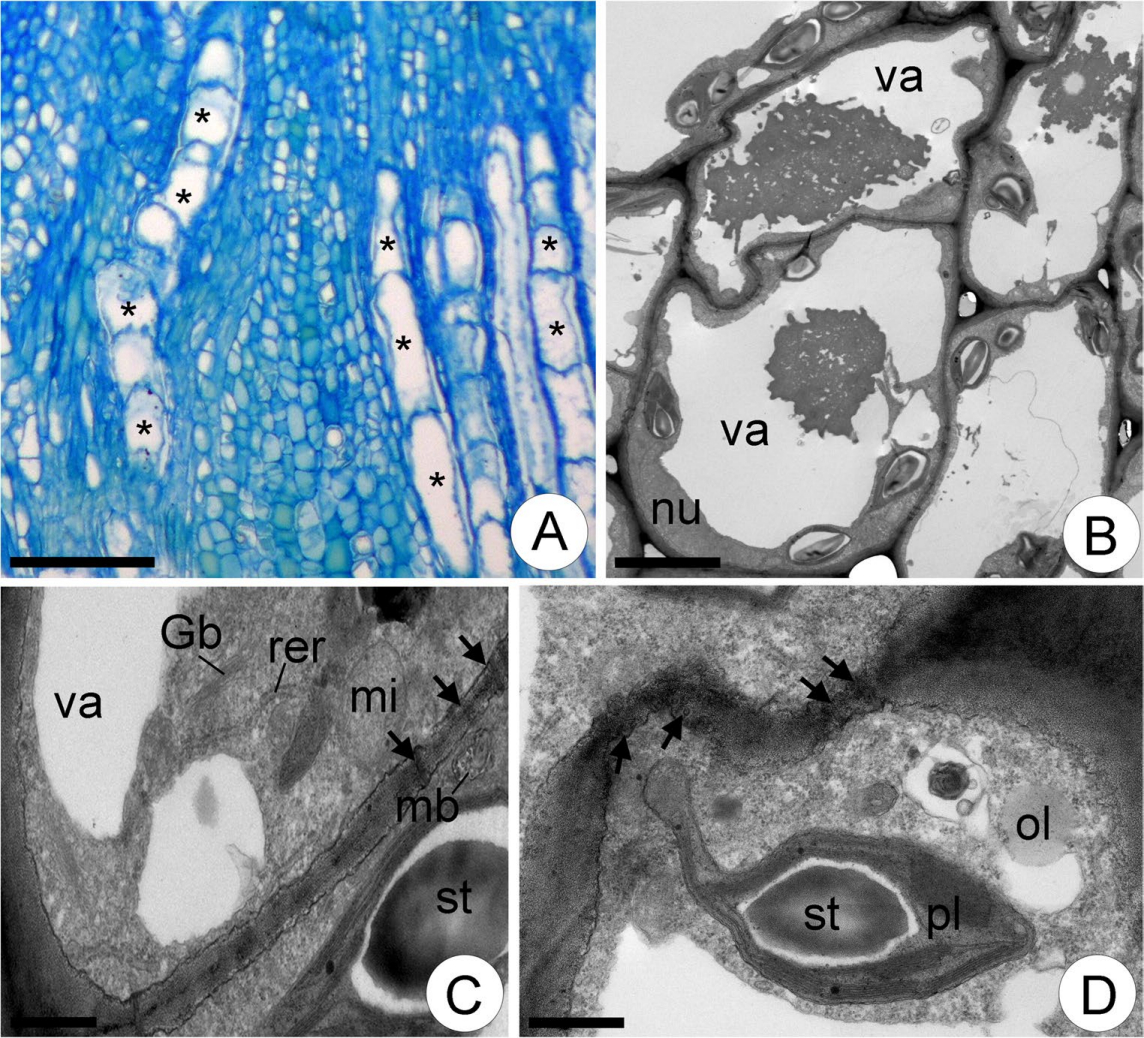
Light (**A**) and transmission electron (**B-D**) micrographs of *Mimosa caesalpiniifolia* laticifer cells in developmental stage 2. **A** Slightly elongated laticifer cells (*) in longitudinal section of young stem stained with toluidine blue. **B** Laticifer cells with large vacuoles, and reduced cytoplasm and nucleus located at the periphery of the cell. **C** Portions of adjacent laticifer cells connected by plasmodesmata. **D** Plastid with protrusion in laticifer cell. Gb: Golgi body, mb: multivesicular boy, mi: mitochondria, nu: nucleus, ol: oil, pl: plastid, rer: rough endoplasmic reticulum, st: starch grain, va: vacuole. The arrows indicate plasmodesmata. Scale bars: A = 100 μm; B = 5 μm; C = 2 μm; D = 1 μm

The stage 2 was characterized by the onset of axial elongation and accumulation of secretion inside vacuoles. In this developmental stage, the laticifer cells began to exhibit a slightly elongated shape in the axial direction of the organs (Fig. 4A). The cell lumen appeared less dense (Fig. 4A, B), the cytoplasm was shrunken, and the nucleus was located at the periphery of the cell (Fig. 4B). Large vacuoles accumulated flocculant material (Fig. 4B). Mitochondria (Fig. 4C) and plastids containing starch grains (Fig. 4B-D) became abundant. Some of the plastids exhibited protrusions (Fig. 4D). Golgi bodies, rough endoplasmic reticulum, free ribosomes, small vesicles and lipids were scattered throughout the cytoplasm (Fig. 4C, D). Multivesicular bodies continue to be detected in these cells (Fig. 4C). Adjacent laticifer cells were connected by large plasmodesmata (Fig. 4C, D).

**Fig. 5:**
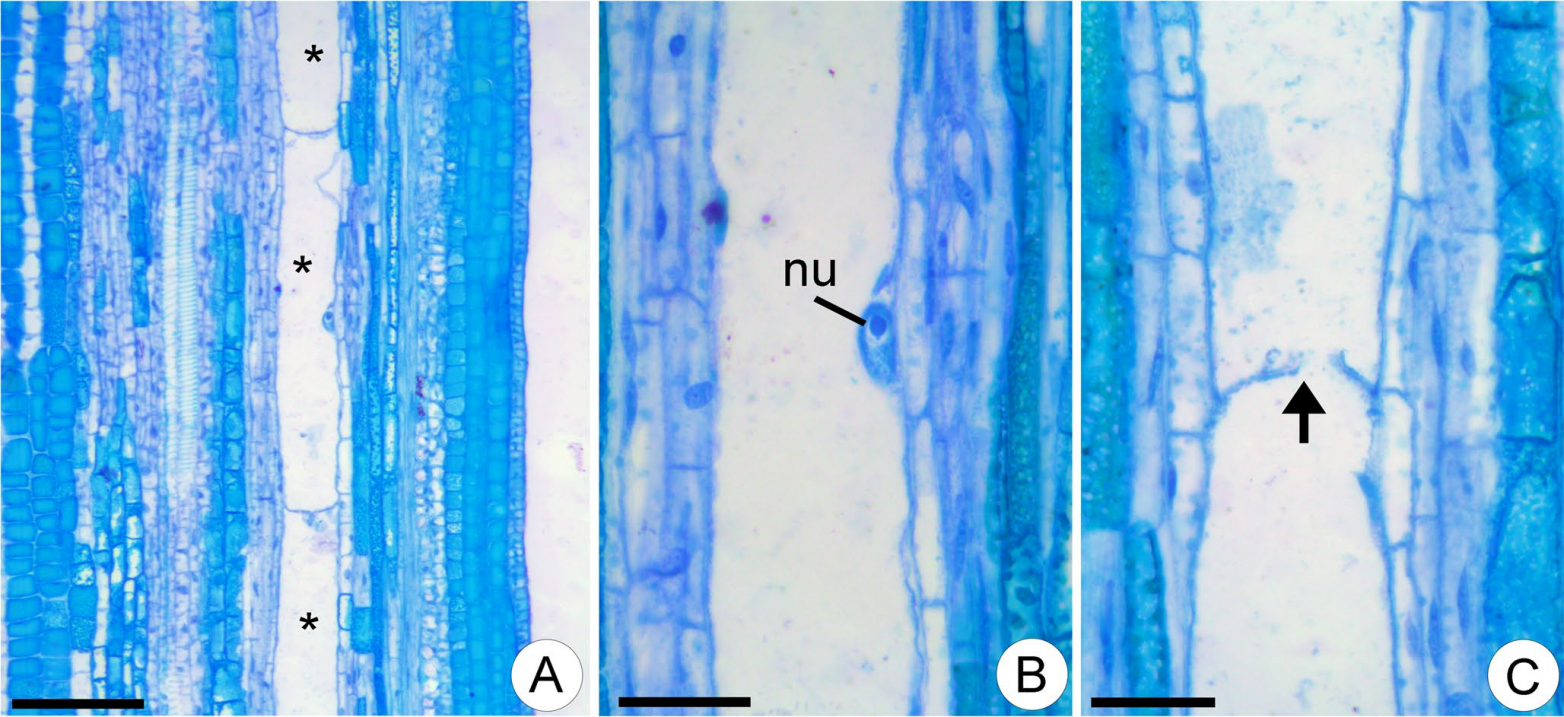
Light micrographs of longitudinal sections of *Mimosa caesalpiniifolia* stem stained with toluidine blue showing laticifer cells in developmental stage 3. **A** Elongated laticifer cells (*) in row showing reduced cytoplasm and thin terminal walls. **B** Detail of laticifer cells exhibiting nucleus confined to the cell periphery. **C** Rupture of the terminal wall (arrow) in a laticifer cells. nu: nucleus. Scale bars: A = 100 μm; B, C = 20 μm

The stage 3 was characterized by the fusion of rowed laticifer cells. In this stage, laticifer cells were elongated, and occurred in differentiated portions of stems (Fig. 5A). Their cytoplasm was reduced to a thin parietal layer and the nucleus remained confined to the cell periphery (Fig. 5A, B). As maturation progressed, the terminal walls of laticifer cells were disrupted (Fig. 5C).

**Fig. 6:**
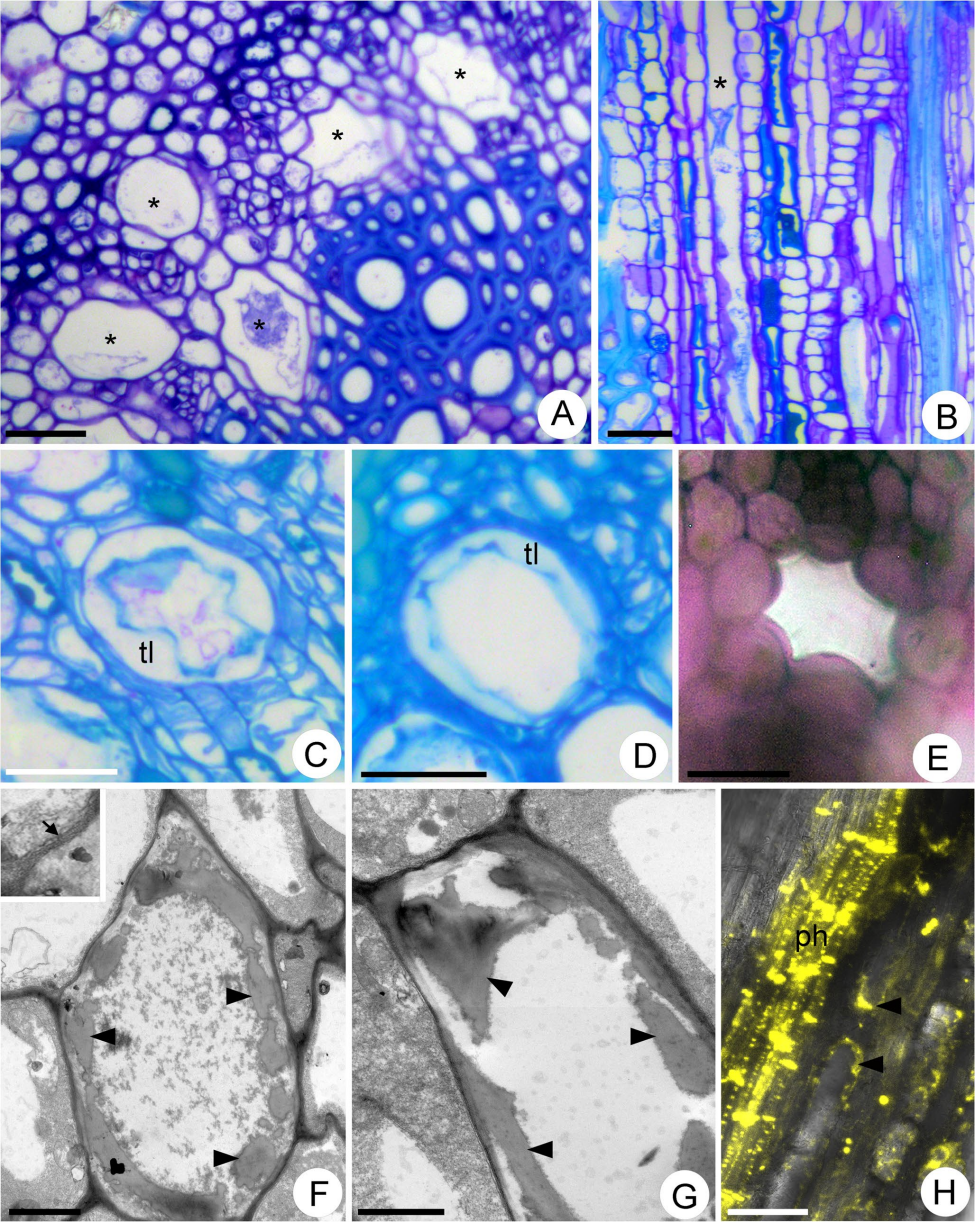
Light (**A-E**,** H**) and transmission electron (**F**,** G**) micrographs laticifer cells in developmental stage 4 in the stem of *Mimosa cesalpiniifolia*. **A** Mature laticifers (*) in cross section stained with toluidine blue. **B** Laticifer (*) in longitudinal section stained with toluidine blue. **C**,** D** Detail of laticifer in cross sections displaying walls with a denser layer strongly stained with toluidine blue, and a thicker layer with a translucent (tl) appearance. **E** Laticifer treated with PAS reagent. **F**,** G** Laticifer cells exhibiting a discontinuous inner parietal portion. Observe plasmodesmata (arrow) in the insert. **H** Longitudinal section of stem treated with aniline blue reagent evidencing the presence of callose (arrows) in the laticifer walls under confocal microscopy. ph: phloem. Scale bars: A = 50 μm; B = 100 μm; C, D = 20 μm; E, H = 30 μm; F, G = 2 μm

Mature laticifers at developmental stage 4 appeared as voluminous, rounded to oval cells in cross section (Fig. 6A), and very long structures in longitudinal view (Fig. 6B). The outer margin of laticifers was slightly sinuous to undulating (Fig. 6A). Their walls varied in thickness (Fig. 6A, C, D) and slight plasmodesmata occurred. Two distinct layers were evident in the cell wall: a denser outer pectin-cellulosic layer strongly stained with toluidine blue, and a thicker and sinuous inner layer with a translucent appearance (Fig. 6C, D). Both cell layers reacted positively to the PAS test (Fig. 6E), indicating their polysaccharidic nature. The inner parietal portion was shown to be a discontinuous layer constituted of homogeneous-looking material under transmission electron microscopy (Fig. 6F, G). The histochemical test using aniline blue reagent observed under confocal microscope confirmed the presence of callose in these walls (Fig. 6H). The protoplasts were filled with latex and had a flocculent appearance (Fig. 6A-C, F). Total lipids, polysaccharides, starch grains, proteins, alkaloids, phenolic compounds, terpenes, and rubber were histochemically detected in the protoplasts of mature laticifers in primary growth stem and fully expanded leaves (Table 1).

**Table 1:** Histochemical tests in mature laticifers in primary growing stem and expanded leaves of *Mimosa caesalpiniifolia* Benth. (Fabaceae)

Reagent	Substances	Presence/absence
Sudan IV	total lipids	+
Ruthenium Red	polysaccharides and pectin	+
Lugol’s reagent	starch grains	+
Ferric chloride	phenolic substances	+
Acidified phloroglucinol	lignin	-
Bromophenol Mercury Blue	protein	+
Nadi reagent	terpenes/oil-resin	+
Periodic Acid/Schiff Reagent (PAS)	neutral polysaccharides	+
Wagner’s reagent	alkaloids	+
Tannic acid/Iron III chloride	mucilage	-
Oil Red O	rubber particles	+
Aniline blue	callose	+

+ : presence ; - : absence

## Discussion

Our study revealed that laticifers in *M. caesalpiniifolia* are articulated nonanastomosing, composed of cells filled with a white, milky emulsion. Large laticifer cells were observed in the phloem across the plant body at the embryonic, seedling, and adult plant stages, forming a continuous laticifer system. The widespread distribution, abundance, and large size of laticifers in *M. caesalpiniifolia* suggest a high investment in latex production, which is thought to play a key role in plant defense, especially in safeguarding aerial organs against herbivores and other natural enemies (Ramos et al. 2019; Freitas et al. 2024). The seedling stage is the most critical stage, when it is most vulnerable to adverse environmental factors, as has been observed for other latex-producing species (Canaveze and Machado 2016). Likewise, we hypothesize that the presence of laticifers in the primary growing organs of *M. caesalpiniifolia* along with their relative scarcity in portions of the plant body during secondary growth may reflect the possibly higher requirement for protection in younger tissues against herbivory. Indeed, the presence of lytic enzymes in latex is thought to be one of the factors contributing to its potential role against biotic agents (Ramos et al. 2019). Studies have suggested a differential role between laticifers immersed in the young portions of the plant body and laticifers in older portions under secondary growth. In *Hevea brasiliensis*, the rubber-tree, Tan et al. (2017) showed that genes related to defense against biotic stresses and rubber biosynthesis were strongly activated in primary laticifers, indicating that these tissues are better prepared to face attacks of biological origin. Conversely, genes associated with abiotic stress and dormancy showed greater expression in secondary laticifers, suggesting that they are better adapted to adverse abiotic conditions (Tan et al. 2017).

Articulated laticifers are characterized by the occurrence of elongated and overlapping cells (Mauseth 1978; Fahn 1979; Medina et al. 2022). In this type of laticifers, the terminal walls that delimit each cell in the row can remain intact, become perforated, or be completely removed during development (Evert 2006), as observed in *M. caesalpiniifolia*. In fact, the rupture of the end walls of the laticifer cells in this species gives rise to tube-like structures filled with latex, as described for articulated laticifers (Evert 2006). Based on our findings, the articulated laticifers of *M. caesalpiniifolia* are nonanastomosing due to the absence of lateral connections between laticifers (Evert 2006). Furthermore, as no branching was observed, the laticifers of *M. caesalpiniifolia* can be classified as unbranched (Fahn 1979; Evert 2006).

Laticifer development in *M. caesalpiniifolia* is initiated in the stem apex and subsequently progresses from the procambial region to the cambium in the secondary stem. Cells destined to differentiate into laticifers exhibited increased cellular activity, as evidenced by the abundance of organelles and cytoplasmic inclusions. High occurrence of mitochondria and plastids containing starch grains characterizes the early developmental stages of the laticifer cells. These cellular alterations, also described in laticifers of other species (Gonçalves et al. 2018), signify an elevated degree of metabolic activity essential for the production of latex and other secondary metabolites. As laticifers develop, cells come to exhibit a reduced cytoplasm resultant from the enlargement of the vacuome. Vacuolar enlargement during laticifer development has been associated with the progressive incorporation of secretory and lytic substances via small vacuoles and vesicles that fuse with the central vacuole (Gama et al. 2017). Previous studies have reported a sequential lysis of cellular components in laticifers, accompanied by the persistence of a thin peripheral cytoplasmic layer surrounding a prominent central vacuole (Stockstill and Nessler 1986; Gama et al. 2017). Such vacuoles have been interpreted as having an autophagic nature (Cai et al. 2009; Zhang et al. 2018). The confinement of the cytoplasm to a thin peripheral layer, along with the pronounced development of the vacuole, is indicative of functional maturity in laticifers of *M. caesalpiniifolia*.

Laticifer cells with digitiform projections with irregularly thickened walls can be evidence of intrusive growth. Intrusive growth occurs when part of the cell maintains contact with neighboring cells while tips of the cell penetrated between adjacent cells (Malhberg 1993), which is associated with subtle adjustments in cytoskeletal organization and in the middle lamella of surrounding cells (Lev-Yadun 2001). The distinctive arrangement of microtubules in the polarized growing regions of the cells facilitates the transport of secretory vesicles, primarily containing pectin (Anderhag et al. 2000), leading to a localized increase in cell wall components. This may explain the formation of irregularly thickened walls in the digitiform projections of laticifer cells in *M. caesalpiniifolia*. The dense cytoplasm and abundance of organelles in these cell portions is remarkable in and can be an indicative of production of enzymes, especially pectinases, necessaries to middle lamellae dissolution necessary for laticifer cell growth among neighboring cells (Mahlberg 1963; Canaveze et al. 2019). In some species with articulated laticifers, laticifer cells appear to exhibit a limited amount of intrusive growth, in which protuberances that developed on these cells intrusively forced their way between the adjacent cells until they came into contact with another laticifer (Malhberg 1993). This situation seems to occur in *M. caesalpiniifolia*.

Multivesicular bodies (MVBs) were noticeable during the early developmental stages of laticifers in *M. caesalpiniifolia*, especially in the stages 1 and 2. MVBs are specialized endosomes containing internal vesicles formed by invagination and budding of the limiting membrane (Cui et al. 2016). A recent study (Petersen et al. 2024) had shown that the plant MVBs are associated with autophagy through several mechanisms or pathways. In the context of intrusive growth, such as in laticifers, MVBs have been suggested to be associated with processes such protein sorting, degradation, and release of extracellular vehicles containing hydrolases. The pectin-rich middle lamella of the laticifer projections may be involved in intrusive growth into adjacent regions. This layer could potentially facilitate the movement of nutrients and water through the cell walls while minimizing friction, which could help prevent cellular damage during laticifer penetration (Serpe et al. 2002). These growth patterns suggest that laticifers have evolved specialized mechanisms to effectively navigate their environments. In addition, the presence of MVBs in contact with the transverse walls of developing laticifer cells in *M. caesalpiniifolia* suggest their involvement in the release of enzymes involved in the breakage of these walls. In fact, the involvement of MVBs in the packing and the secretion of hydrolytic enzyme has been reported during the process of wall degradation and microfibril reorientation (Zamski et al. 1983), including in laticifer development (Gama et al. 2017).

Total lipids, acid polysaccharides, terpenes, and rubber particles were detected histochemically in the protoplasts of mature laticifers in stem and leaves of *M. caesalpiniifolia*. Essentially, latex corresponds to the modified protoplast of laticifer cells (Matile 1987; Freitas et al. 2024) and contains a wide variety of substances in solution and colloidal suspensions, including carbohydrates, organic acids, tannins, lipids, terpenes, rubbers, and other substances in liquid fluid, which vary in composition, color, and refractive index in different taxa. In general, these metabolites are believed to contribute to plant defense against adverse factors, particularly herbivorous insects (Hagel et al. 2008).

Although the occurrence of plasmodesmata in mature laticifer cells is a controversial issue (Pickard 2008), such intercellular communication structures were here visualized in *M. caesalpiniifolia* laticifers, including mature laticifers. Studies have indicated the occurrence of non-functional plasmodesmatal connections between laticifer cells and the neighboring tissues (see Pickard 2008 and literature therein), being the breaking of plasmodesmatal connections among laticifers and adjacent cells resultant from the laticifer intrusive growth (Jarves et al. 2003; Canaveze et al. 2019). Laticifer cells are thought to derive energy from the apoplast (Pickard 2008), with their loading involving symplastic transport of photoassimilates and minerals from the phloem, followed by apoplastic transfer and uptake into laticifers via a proton–sugar symporter, where these compounds may be converted into latex (Pickard 2008; Bouteau et al. 1999; Abarca et al. 2019). In *M. caesalpiniifolia*, although plasmodesmata were clearly observed, their functional status cannot be determined. However, the presence of callose in these cells may potentially interfere with symplastic transport through plasmodesmata, as discussed below.

The presence of callose in the walls of laticifer cells *of M. caesalpiniifolia* is a remarkable feature in this study. Callose has been reported in laticifers of different plant species, such as those belonging to Asteraceae (Vertres and Mahlberg 1978), Apocynaceae (Canaveze et al. 2019) and Sapindaceae (Medina et al. 2021). However, to the best of our knowledge this represents the first documented evidence of callose in laticifers of a legume species. The role of callose in organ development and in plant environmental responses is well recognized (Amsbury et al. 2018). Callose performs multiple functions during different developmental processes in plants encompassing support and defense mechanisms (Piršelová and Matušíková 2013). It is generally accepted that callose provides mechanical support to plasmalemma and/or to the cell wall against different environmental stresses including injury, cold, low temperature, heavy metals and various pathogens (Luna et al. 2011). While callose plays a role in normal development, it is also a key component of the plant’s defense response to wounds and pathogens. Callose deposition serves as a sealant, creating a physical barrier to protect the wound site. In laticifers, callose contributes to the initial seal at the wound site, facilitating the solidification of latex and preventing its movement toward nearby cells during the healing process (Wang et al. 2022). This information is consistent with observations in *M. caesalpiniifolia*, in which callose is deposited in mature laticifer cells and may lead to the obliteration of plasmodesmata that persist into this developmental stage. Indeed, callose is thought to regulate the movement of molecules through plasmodesmata, serving as a developmental regulator of symplasmic continuity (Benitez-Alfonso 2014). Callose deposition at laticifer cell junctions temporarily regulates plasmodesmatal connectivity promoting momentary isolation and allowing controlled cell growth, fusion, and protoplast reorganization. Callose may control the movement of proteins, RNAs, and other molecules during differentiation by changing the size exclusion limit of plasmodesmata (Xu and Jackson 2010; Amsbury et al. 2018). Despite the significant progress made in understanding the role of the callose in the plant cell walls (Amsbury et al. 2018), many questions remain regarding its synthesis and its involvement in defense responses in laticifer. This study underscores promising research avenues centered on the synthesis and localization of callose within laticifer walls, as well as its functional significance in this secretory system. Additionally, understanding the molecular mechanisms that govern callose deposition may yield valuable insights into plant resilience and adaptive responses.

In summary, our results demonstrate that laticifers are distributed throughout the vegetative plant body from the earliest developmental stages, forming a continuous system across the successive phases of vegetative growth in *Mimosa caesalpiniifolia*. Moreover, we identified a diverse array of chemical compounds within these cells. To our knowledge, this study represents the first report of callose deposition in laticifers of legume species and may represent an important mechanism for maintaining the structural integrity and functional efficiency of laticifers. Overall, this finding advances the understanding of laticifer development and uncovers previously unrecognized regulatory processes in legume secretory systems. This study fills a significant gap in the understanding of the origin and typology of laticifers in Fabaceae and provides valuable insights to support taxonomic, ecological, and sustainable-use studies of this multipurpose forest species native to the Brazilian semi-arid region.

## Data Availability

All data generated or analyzed during this study are included in this published article.
